# *In Vitro* Prevention of *Salmonella* Lipopolysaccharide-Induced Damages in Epithelial Barrier Function by Various *Lactobacillus* Strains

**DOI:** 10.1155/2013/973209

**Published:** 2013-06-06

**Authors:** Chun-Yan Yeung, Jen-Shiu Chiang Chiau, Wai-Tao Chan, Chun-Bin Jiang, Mei-Lien Cheng, Hsuan-Liang Liu, Hung-Chang Lee

**Affiliations:** ^1^Division of Gastroenterology and Nutrition, Department of Paediatrics, Mackay Memorial Hospital, Taipei 10449, Taiwan; ^2^Mackay Medicine, Nursing and Management College, Taipei 25245, Taiwan; ^3^Institute of Biotechnology and Department of Chemical Engineering, National Taipei University of Technology, No. 1, Sec. 3, Chung-Hsiao East Road, Taipei 10608, Taiwan; ^4^Department of Medicine, Mackay Medical College, Taipei 25246, Taiwan; ^5^Department of Medical Research, Mackay Memorial Hospital, Taipei 25160, Taiwan; ^6^Division of Gastroenterology and Nutrition, Department of Paediatrics, Mackay Memorial Hospital, No. 690, Sec. 2, Guangfu Road, Hsinchu 30071, Taiwan; ^7^Department of Paediatrics, Taipei Medical University, Taipei 11031, Taiwan

## Abstract

*Background*. *Lactobacillus* shows beneficial anti-inflammatory effects on *Salmonella* infection. The maintenance of the tight junction (TJ) integrity plays an importance role in avoiding bacterial invasion. Whether *Lactobacillus* could be used to regulate the TJ protein expression and distribution in inflamed intestinal epithelial cells was determined. *Methods*. Using the transwell coculture model, *Salmonella* lipopolysaccharide (LPS) was apically added to polarized Caco-2 cells cocultured with peripheral blood mononuclear cells in the basolateral compartment. LPS-stimulated Caco-2 cells were incubated with various *Lactobacillus* strains. TJ integrity was determined by measuring transepithelial electrical resistance across Caco-2 monolayer. Expression and localization of TJ proteins (zonula-occludens- (ZO-) 1) were determined by Western blot and immunofluorescence microscopy. *Results*. Various strains of *Lactobacillus* were responsible for the different modulations of cell layer integrity. LPS was specifically able to disrupt epithelial barrier and change the location of ZO-1. Our data demonstrate that *Lactobacillus* could attenuate the barrier disruption of intestinal epithelial cells caused by *Salmonella* LPS administration. We showed that *Lactobacillus* strains are associated with the maintenance of the tight junction integrity and appearance. *Conclusion*. In this study we provide insight that live probiotics could improve epithelial barrier properties and this may explain the potential mechanism behind their beneficial effect *in vivo*.

## 1. Introduction 

*Salmonella *infection is a common cause of human food poisoning worldwide and can induce a broad spectrum of diseases from mild diarrhea to typhoid. All *Salmonella* serotypes share the ability to gain entry to the host through oral ingestion of contaminated food or water. They induce their own uptake into intestinal epithelial by *Salmonella* pathogenicity island-1 (SPI-1) type 3 secretion system. This epithelial barrier function can be further weakened by infection with bacteria, including *S. typhimurium *effectors [1, 2]. *Salmonella enterica* serovar Typhimurium has developed means of breaching the mucosal epithelial barrier by usurping signaling mechanisms within host cells [3]. It is likely that *Salmonella* induces localized effects on tight junction permeability during intestinal infections. These effects may act synergistically with other conditions, such as inflammatory responses, to promote tight junction (TJ) dysfunction [4]. At present, *S. typhimurium* overcoming the intestinal barrier is the most widely accepted mechanism for their entering into host cells, especially deeper tissues. Thus, the maintenance of the tight junction integrity of polarized epithelial monolayer plays an important role in avoiding bacterial invasion.

Besides the disrupted barrier function by* S. typhimurium* effectors, evidence also showed that lipopolysaccharide (LPS) could induce derangements in intestinal epithelial barrier function. In our previous study, we demonstrated the evidence of *Salmonella *LPS-induced inflammation and epithelial barrier dysfunction [5]. However, the specific TJ responsible for the barrier dysfunction has not been identified. By using polarized epithelial monolayers, we might clarify the mechanism that *Salmonella *LPS alters the distribution of TJ protein. 

Regarding the clinical treatment of Salmonellosis, conservative and supportive care remains the principal standard of management. Antibiotics are only reserved for invasive diseases and patients with toxic manifestations. Routine antibiotics usage appears to increase the risk of emergence of multidrugs resistant *S. typhimurium* strains [6]. The patients at risk of developing multidrug resistant bacteria would benefit from prophylactic therapy in the past decade. However, lack of an effective antibiotic for treatment might promote multidrugs resistant bacteria resulting in excess morbidity and mortality [7].

The potential improvements by using probiotics in intestinal epithelial barrier function after *Salmonella* infection have been demonstrated increasingly both in clinical trials and experimental models. In animal models, probiotic mixtures had been used to ameliorate diarrhoea in *S. typhimurium*-infected pigs [8]. Oral administration of VSL#3 was also shown to be effective as a primary therapy in mice. Those probiotic mixtures were thought to have an effect in enhancing barrier integrity by directly altering the epithelial permeability and thus protecting the epithelial cells from pathogenic bacterial invasion [7].

We hypothesize that *Lactobacillus* is able to regulate the TJ protein expression and distribution in inflamed intestinal epithelial cells and hence change TJ structure. In our previous study [5], we tested the interaction of *S. typhimurium* LPS in a cultured polarized human epithelial cell model. We successfully demonstrated that *S. typhimurium *LPS could disrupt TJ structure in epithelial monolayer while examining changes in resistance and cell permeability. We also investigated TJ protein expression such as zonula-occludens- (ZO-) 1 acting as adapters at the cytoplasmic surface of TJ [9], as well as the* in vitro *effects of *Lactobacilli* on TJ protein distribution. Our findings suggested an important role of *Lactobacillus* in regulating the structure and function of TJ in intestinal cells. In this study, we tried to determine the potential abilities of various *Lactobacillus* strains in enforcing the epithelial cell barrier in response to the enteric pathogen *S. typhimurium *LPS challenge.

## 2. Materials and Methods

### 2.1. Bacteria and Culture Conditions

*Lactobacillus rhamnosus *GG (LGG) was purchased from BCRC (Bioresource Collection and Research Center, Taiwan). The commercial strain *L. casei variety rhamnosus, *Lcr35 (Antibiophilus), was used in this study. *L. rhamnosus *(LR), *L. paracasei* (LP), *L. johnsonii* 50 (LJ50), and *L. johnsonii* 59 (LJ59) were obtained from TTY Biopharm (Taiwan). Those *Lactobacilli* were grown under limited aeration at 37°C in MRS medium (Difco). The number of live bacteria colony forming unit (CFU) was deduced from the absorbance at 600 nm (A600), using a calibration curve for each strain. Bacterial cells were grown till stationary phase, washed twice in sterile phosphate buffered saline (PBS, pH 7.2), resuspended at 1 × 10^9^ CFU/mL in PBS containing 20% glycerol, and stored at −80°C until further use. 

### 2.2. Cell Culture and Caco-2/Human Blood Peripheral Monocyte Cells (PBMC) Coculture Model

Human colon adenocarcinoma Caco-2 cells (Bioresource Collection and Research Center, BCRC 67001; Taiwan; passage 33–37) were grown in 0.8 mL Dulbecco's modified Eagle medium (DMEM) supplemented with 2.0 mM L-glutamine, 0.1 mM nonessential amino acids, 10 mM NaHCO_3_, 1.7 mM glutamine, 1.0 mM sodium pyruvate, and 20% (v/v) fetal bovine serum (ASFC Bioscience, Australia). Cells were seeded 1 × 10^5^ cells/cm^2^ in 12-well transwell inserts (CORNING, 0.4 *μ*m). PBMCs were used from at least three blood donors and isolated by using Ficoll-Hypaque (GE Healthcare Bio-Science AB, Sweden) gradient centrifugation from healthy donors. PBMCs were (4.0 × 10^6^) transferred to tissue culture plates and were cultured in 1 mL RPMI 1640 containing 10% serum. Cell cultures were maintained at 37°C in 95% air and 5% CO_2_ in a humidified atmosphere with three cell culture medium changes per week.

### 2.3. Induction Inflammatory Response and Anti-Inflammation Scoring

To induce the inflammatory response, we added *S. typhimurium *LPS (10 ng/mL, L6143, Sigma) to the apical compartment on the basis of preliminary time-course studies [5]. Negative control group had no LPS treatment. After 3 hr of stimulation of positive control group, all of the culture media in both compartments were removed and washed twice with cold PBS. Inflamed polarized Caco-2 monolayer was added with fresh medium containing various *Lactobacillus *strains or positive control group without* Lactobacillus*, for 1, 6, and 24 hr. 

### 2.4. Electrical Resistance Measurements

Confluence was controlled by measurement of the transepithelial electrical resistance (TEER; World Precision Instruments, Sarasota, FL, USA). Cut-off point of TEER was defined as 450 Ω  × cm^2^ and we also visualized the cell layer integrity under the microscope for consistency. TEER was read at four time points (0, 1, 6, and 24 hr).

### 2.5. Immunofluorescence Microscopy

For immunofluorescence study, polarized Caco-2 monolayers were plated onto chamber glass slides (Deckglaser) and washed twice with PBS 3 hr prior to the exposure to LPS (10 ng mL^−1^). *Lactobacillus* was added into the inflamed monolayers and incubated for 24 hrs. Cells were then fixed in 4% paraformaldehyde (Merck) and permeabilized with 0.3% Triton X-100 (Sigma-Aldrich). To study the TJ, unspecific bindings were blocked with 5% fetal bovine serum (SAFC, Australia) 45 min prior to the incubation with primary antibody (1 : 300 mouse anti-tight junction protein-1 (anti-ZO-1) 0.1 mg/mL; Sigma, USA) for 60 min and secondary antibody (1 : 500 Dylight 488 goat antirabbit immunoglobulin G; Jackson, USA) for 30 min. The results were analyzed using a BX60 fluorescence microscope (Olympus, Hamburg, Germany). ZO-1 staining was observed blindly by two individual observers in duplicate samples and performed three times.

### 2.6. Western Blot

Inflamed polarized Caco-2 monolayers were added *Lactobacillus* and grown on 24-wells plate. They were harvested after incubation for 24 hr. Individual samples were lysed in an ice-cold lysis buffer (20 mM Tris-HCl, pH 7.4; 150 mM NaCl; 5 mM MgCl_2_; 10% glycerol; 0.5% NP-40; 0.1% SDS; 0.3 *μ*M aprotinin; 1 *μ*M leupeptin; 1 mM PMSF; 1 *μ*M pepstatin A) and placed on ice for 10 minutes. The lysed samples were centrifuged at 10,000 rpm for 5 minutes at 4°C and the supernatant was collected. Total protein concentration was quantified by use of the BCA protein assay kit (Thermo, USA). For protein analysis, 40 *μ*g of protein was added to an equal volume of 2 × Laemmli sample buffer and boiled for 10 minutes. The samples were run at 8% polyacrylamide gel at 100 V for 1.5 hr. Protein was transferred to Immunoblot PVDF membranes (Bio-Rad). After overnight blocking (PBS/Tween supplemented with 0.05% nonfat dry milk), blots were incubated with primary (mouse anti-tight junction protein-1 (anti-ZO-1) (Sigma, USA)) and secondary antibodies (horseradish peroxidase conjugated anti-mouse IgG (Jackson, USA)) for 60 minutes at room temperature; proteins were visualized by chemiluminescence reagents (Thermo, USA) and exposed to X-OMAT film (Kodak, USA). 

*Statistics.*  The quantitative data were expressed as mean ± standard error (SE) for triplicate measurements. Statistical analyses were performed with Student's *t*-test using SPSS 12.0. Statistical significance was defined as a *P* value of <0.05.

## 3. Results

### 3.1. Effect of *Lactobacillus* on Caco-2 Monolayer Resistance

**Figure 1:**
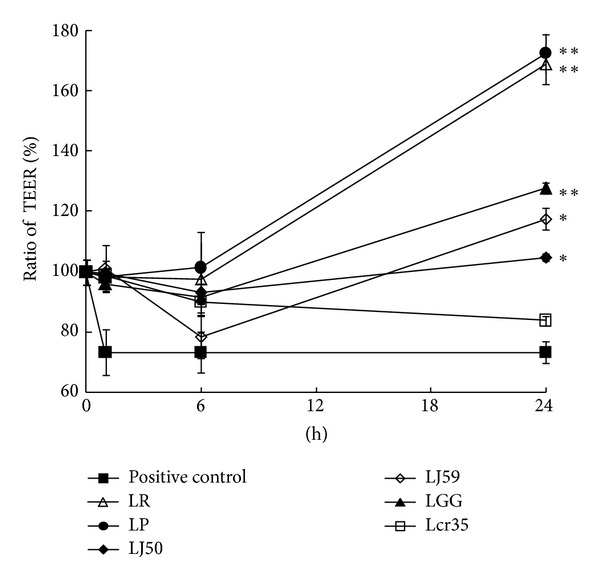
Effect of Lactobacilli on Caco-2 monolayer resistance. TEER ratio of inflamed Caco-2 monolayers exposed to positive control medium (■), LGG (▲), Lcr35 (□), LR (△), LP (●), LJ50 (◆), and LJ59 (⋄) into the apical compartment. TEER (Ω  × cm^2^) was expressed as the percentage at time 3 hr in relation to the initial value for each treatment. *P < 0.05; **P < 0.005.

To determine whether the alterations in Caco-2 monolayer resistance required the direct effects between the *Lactobacillu*s and intestinal epithelial cells, we selected six strains of* Lactobacillus* and exposed them to polarized Caco-2 monolayer individually. Significant differences in the measured values for the polarized monolayer were observed 3 hr after LPS treatment. TEER was a measurement of the integrity of the epithelial barrier and was monitored over the course of a 24 hr protection. We found that the TEER values from cultured epithelial cells in positive control group were consistently the lowest at each time frame (Figure 1). TEER values of various *Lactobacillus *groups remained relatively close 1 hr after incubation. Most *Lactobacillus *strains did not induce a significant increase of TEER 6 hr later. However, after 24 hr incubation, TEER values in LP and LR groups were found to have the highest levels among all *Lactobacillus* groups. TEER levels of Lcr35 group remained the lowest through the 24 hr period. TEER values in LGG, LJ50, and LJ59 groups were lower than those of LP and LR groups and had statistically significant differences. These results suggested that various strains of* Lactobacillus* were responsible for the different modulations of cell layer integrity.

### 3.2. Effects of LPS on ZO-1 Location and Expression

**Figure 2:**
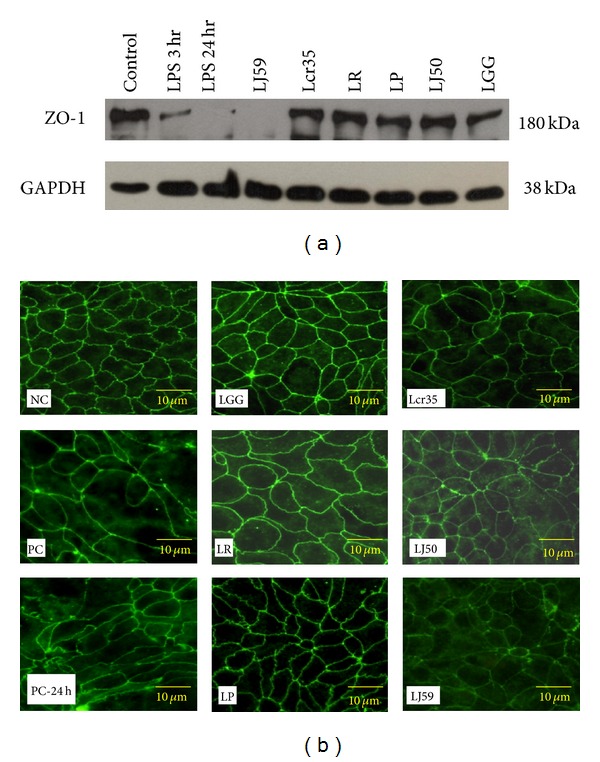
The appearances of tight junctions were evinced by immunostaining ZO-1 expression and localization after LPS and *Lactobacillus* administration. Negative control (NC) without treatment and positive control (PC) exposed to LPS for 3 hr were shown. Inflamed Caco-2 cells were colonized without bacteria (PC-24 h) or with LGG, LR, LP, Lcr35, LJ50, or LJ59 for 24 hr. (a) Representative 180-kDa ZO-1 band was shown by Western blot analyses. Expression of ZO-1 showed significantly lower levels in the PC and PC-24 h groups when compared to the NC group. (b) Representative slides were evaluated by immunofluorescence microscopy. Expression of ZO-1 showed similar levels in the LR, LP, and LGG groups when compared to the NC group. GAPDH: glyceraldehyde 3-phosphate dehydrogenase.

As TJ was crucial for the barrier function, the effects of *Lactobacillus* on the TJ integrity were further studied. To determine the expression of ZO-1 on LPS-induced disruption of TJ, we performed the Western blot analysis and found that the TJ membrane protein ZO-1 was markedly degraded in PC and PC-24 h groups (Figure 2(a)). We also used TJ marker including ZO-1 for immunofluorescence microscopy study on colon epithelium (Figure 2(b)). ZO-1 distribution in the negative control group cells had its normally smooth nature. However, ZO-1 was highly disrupted in epithelial cells exposed to LPS, appearing in a discontinuous bead-like pattern in the LPS treatment group. The 3 hr exposure to LPS resulted in loss of ZO-1 at the intercellular junctions compared to the negative control group. LPS was found to be involved in the disruption of epithelial barrier and disrupted the location of ZO-1. 

### 3.3. Effects of *Lactobacillus* on ZO-1 Location and Expression Exposed to LPS

*Lactobacillus *plays an important role in the maintenance of epithelial barrier [1]. Our Western blot analysis revealed a significant increase of ZO-1 expression in various *Lactobacillus* strains and showed similar densities except in PC, PC-24 h, and LJ59 groups (Figure 2(a)). In addition, expression of ZO-1 showed similar levels in the LR, LP and LGG groups when compared to the NC group. We found that these* Lactobacilli* strains were also responsible for the changes in the location of TJ protein. Polarized Caco-2 monolayers exposed to LPS were incubated with various strains of *Lactobacillus* for 24 hr, and the localization of ZO-1 was examined via microscopy (Figure 2(b)). The disrupted TJ of negative control group could not be reversed after 24 hr incubation. In negative control, strains of LGG and Lcr35 had no effect on TJ structure, appearance, and size. Besides, exposure to LR also caused the TJ structure to curve markedly, and ZO-1 became larger in appearance. In negative control and LP-incubated cells groups, ZO-1 was found to be localized to the lateral cell membrane and formed a characteristic continuous web-like pattern. Moreover, we found cloudy web-like ZO-1 appearance in the groups with strains LJ 50 and LJ 59 treatments. Even after the removal of LPS for 24 hr, we found the appearance of ZO-1 remained the same. As a conclusion, we showed here that the disrupted TJ could not be reversed at limited period after the removal of LPS. However, lactic acid bacteria strains could be able to conserve the appearance of the TJ entirely. 

## 4. Discussion

In human beings, the mucosal barrier of the gastrointestinal tract is composed of the intestinal microbial flora, the mucus layer, the epithelial cells, and the intercellular TJ positioned between them [10]. TJ are the key molecules involved in the control of paracellular permeability [11, 12]. TJ are complex protein structures comprised of transmembrane proteins, which interact with the actin cytoskeleton via plaque proteins. Signaling pathways involved in the assembly, disassembly, and maintenance of TJ are controlled by a number of signaling molecules, such as protein kinase C, mitogen-activated protein kinases, myosin light chain kinase, and Rho GTPases [13]. They seal the paracellular space between epithelial cells, thus preventing paracellular diffusion of microorganisms and other antigens across the epithelium. 

Commensal bacteria, which normally colonize the murine gut during the first several weeks of postnatal life, induce expression of genes that improve intestinal barrier function, whereas abnormal bacterial colonization may disrupt this process and contribute to the development of host diseases [14]. Commensal probiotics have been shown to promote intestinal barrier integrity both *in vitro* and *in vivo*. Probiotics preserve the intestinal barrier in mouse models of colitis [8] and reduce intestinal permeability in human patients with Crohn's Disease [15]. Treatment of epithelial cells with *Escherichia coli* Nissle 1917 leads to increase in expression of ZO-2 protein and redistribution of ZO-2 from the cytosol to cell boundaries *in vitro* [16]. Recently, *L. plantarum* DSM 2648 was able to reduce the negative effect of *E. coli* (enteropathogenic *E. coli* (EPEC)) O127:H6 (E2348/69) on TEER and adherence by as much as 98.75% and 80.18%, respectively, during simultaneous or prior coculture compared with EPEC incubation alone [17]. Administration of *L. plantarum* into the duodenum of healthy human volunteers was shown to significantly increase ZO-1 and occludin in the vicinity of TJ structures [18].

In this study we showed that *Lactobacillus* could attenuate the barrier disruption disruption of intestinal epithelial cells caused by *Salmonella *LPS administration. Addition of *Lactobacillus* to the cell culture medium was able to reduce the LPS-induced inhibition of TEER and reverse the change in TJ protein ZO-1 expression. 

Although this present study did not address the mechanism by which probiotics inhibited LPS-induced damage in Caco-2 cells, it could be hypothesized that *E. coli* strain Nissle 1917 (EcN) might do so by restoration of a disrupted epithelial barrier infected by *E. coli* strain E2348/69 [16]. Other studies indicated that the anti-inflammatory effect of live *Lactobacillus acidophilus* on increased transepithelial resistance contrasts markedly with the fall in resistance evoked by *E. coli *infection [19]. The fact that different *Lactobacillus* strains had different varying characteristics in repairing the intestinal barrier might explain why LP was able to recover the LPS-induced damage in polarized Caco-2 monolayer more efficiently than LJ50 and LJ59 did when we looked at their TEER expressions. 

Another conceivable mode of action for lactic acid bacteria is that they directly modulated the function of epithelial cells. The TJ might play a role in preventing the entrance of pathogens into the epithelial cells. It was reported that fermented fruit by probiotics stimulates TJ maintenance and formation [20]. In addition, LGG had the ability to prevent changes in host cell morphology, attaching/effacing lesion formation and monolayer resistance against enterohemorrhagic *E. coli* O157:H7 infection [21]. At least, some probiotics were shown to have the ability of stabilizing TJ and inducing mucin secretion in epithelial cells [22]. After LPS stimulation, coincubation with strain LC could stabilize TJ structure as well. Besides, the addition of strains LGG and Lcr35 also resulted in a similar TJ morphology as original in the Caco-2 monolayer. In contrast to the LJ59 group, TJ structure was more poorly maintained compared to positive control. Thus we found *Lactobacillus* may have the potential to protect the epithelial barrier from damage induced by LPS direct action on the epithelial cells. 

Although in different study settings, previous studies reported that* Lactobacillus *had beneficial anti-inflammatory effects on *Salmonella*  [5], it is still difficult to ascertain their direct mechanism of action. Our previous findings documented the anti-inflammatory effects of Lcr35 *Salmonella* LPS [5]. Several studies showed that administration of different *Lactobacillus* strains could reduce diarrhea [23]. Damaged tight junction and disrupted intestinal barrier occur during enterocolitis. In addition, probiotics administration had shown to have an anti-inflammatory effect and play an important role in treatment of enterocolitis [24].

In conclusion, we provide insight that live probiotics could improve epithelial barrier properties and this may explain the potential mechanism behind their beneficial effect *in vivo*.
